# Heat pain modulation with virtual water during a virtual hand illusion

**DOI:** 10.1038/s41598-019-55407-0

**Published:** 2019-12-13

**Authors:** Ivo Käthner, Thomas Bader, Paul Pauli

**Affiliations:** 1 Department of Psychology I, Biological Psychology, Clinical Psychology and Psychotherapy, University of Würzburg, Würzburg, Germany; 2 Center of Mental Health, Medical Faculty, University of Würzburg, Würzburg, Germany

**Keywords:** Neuroscience, Psychology, Cognitive neuroscience

## Abstract

Immersive virtual reality is a powerful method to modify the environment and thereby influence experience. The present study used a virtual hand illusion and context manipulation in immersive virtual reality to examine top-down modulation of pain. Participants received painful heat stimuli on their forearm and placed an embodied virtual hand (co-located with their real one) under a virtual water tap, which dispensed virtual water under different experimental conditions. We aimed to induce a temperature illusion by a red, blue or white light suggesting warm, cold or no virtual water. In addition, the sense of agency was manipulated by allowing participants to have high or low control over the virtual hand’s movements. Most participants experienced a thermal sensation in response to the virtual water and associated the blue and red light with cool/cold or warm/hot temperatures, respectively. Importantly, the blue light condition reduced and the red light condition increased pain intensity and unpleasantness, both compared to the control condition. The control manipulation influenced the sense of agency, but did not influence pain ratings. The large effects revealed in our study suggest that context effects within an embodied setting in an immersive virtual environment should be considered within VR based pain therapy.

## Introduction

Contextual and cognitive manipulations gain interest in pain research because of apparent analgesic effects without the adverse side effects of pharmacological treatments^1,2^. A repeatedly demonstrated contextual manipulation is the association of the colours red and blue with feelings of warm/hot and cool/cold, respectively^3–6^, which is likely to be a cultural norm^4^. It is omnipresent in daily life, for instance, these colours indicate the temperature on a thermometer or a water tap. This well-established association between colour and temperature can be applied to modulate temperature and pain perception^7–9^. For example, Moseley and Arntz^9^ manipulated the context of a very cold (−20 °C) stimulus applied to the participants’ hand by simultaneously presenting either a blue or a red light. In consequence, the participants perceived the cold stimulus as either cold or warm and less or more painful, respectively.

In the current study, we built on the effects known to alter pain perception to modulate pain within an immersive and embodied virtual reality scenario and investigate factors related to virtual embodiment and pain.

Immersive virtual reality (IVR) allows users to experience a feeling of presence in a computer-simulated world^10–13^. This sense of ‘being there’ in the virtual environment is an important factor for the distractive value of IVR, which generally has strong analgesic effects in acute pain^14–17^ and beneficial effects in chronic pain^18–20^. IVR is a powerful method to modify the environment and context and thereby influence experience. However, the importance of the type of virtual environment is unclear. For example, Mühlberger and colleagues^21^ had participants passively move through a snowy virtual winter landscape versus a predominantly yellow and red autumn landscape, but found similar ameliorating effects on hot or cold pain stimuli.

IVR offers unique possibilities to gain ownership over virtual limbs or an entire virtual body^22–27^. Virtual embodiment changes the interaction with the virtual world profoundly^27^. Further, it allows to modify the virtual body and can be used for pain relief (for a recent review see Matamala-Gomez *et al*.^28^). Prior to the availability of IVR, embodiment was mainly investigated with a now classic paradigm, the so-called Rubber Hand Illusion (RHI)^29^. It demonstrated that the experience of ownership over an artificial body part is possible^29^. In this paradigm, most participants report a feeling of ownership/mineness over a fake rubber hand if they observe it while it is being stroked in synchrony with their real hand that is hidden from sight^30^. Apart from visuotactile stimulation other stimulation patterns, such as visual-thermal can induce the illusion^31^. More recent VR studies built on the RHI to create a virtual hand illusion (VHI)^32–36^. This was achieved via different means, e.g. synchronous visuotactile stimulation similar to the classic RHI^8,34,35,37^, through a motor imagery based brain-computer interface^38^, a virtual hand flashing in synchrony with the own heartbeat^39^, or simply observing a virtual hand co-located with the real hand from a first-person perspective^36^. One such study^8^ successfully altered pain perception by manipulating the colour of the skin of the virtual hand. Participants saw a virtual environment presented through a head-mounted display (HMD). They were seated in the position of a virtual avatar and saw a virtual hand, co-located with their real hand resting on a table in front of them. To induce a VHI the experimenter moved the index finger of the participants and the virtual finger moved accordingly. In the experimental conditions, a coloured spot appeared as soon as the temperature started rising and participants were asked to indicate their heat pain threshold. Either a coloured spot (blue, red or green) appeared at the position of the heat stimulus on the participants arm or a grey spot on the table next to the hand turned into red. The heat pain threshold was significantly lower for the red spot condition compared to the blue spot and this effect was specific for the red spot on the arm as the pain threshold was highest for the red spot displayed on the table. This study demonstrated an influence of skin colour on pain perception with the VHI and that the interaction with the virtual environment might be of particular importance when attempting to modulate pain within IVR.

In none of the previous studies investigating the effects of virtual embodiment on pain^28^, participants were allowed to freely move their hands. In the present study, we induced a VHI by the experience of co-located movements of the own with a virtual hand, i.e. participants actively moved their hands and could, therefore, experience a sense of agency (control over the movements of the virtual hands) and interact with the virtual world more naturally compared with previous studies. There is evidence that matched multisensory information (e.g. synchronous movements of the own hand and an observed virtual object) is sufficient to induce an ownerhip illusion over an artificial object^40^.

Previous studies have investigated the effects of observing movements of a virtual hand, but found no effects on pain^41,42^. To our knowledge, however, no study until now has manipulated the level of control over a virtual hand to influence the sense of agency and investigate its effect on pain perception. The sense of agency was manipulated via a high or low level of control over the virtual hand. We hypothesised that higher agency might lead to a reduced pain experience since it was previously demonstrated that perceived control can lead to a reduction in pain ratings^43^. The effects of virtual contexts on pain perception were examined by applying painful heat stimuli to the participants’ forearm while they placed their virtual hand under a virtual water tap during different context manipulations, i.e. a red, blue or white light on top of the tap, suggesting warm, cold or no water. We expected an analgesic effect of the blue light and an increased pain perception for the red light condition compared with the no water control condition.

The main outcome measures of the study were pain ratings (intensity and unpleasantness) of the study participants along with ratings of sense of agency. We further assessed ownership and sense of self-location as they are the subcomponents in the working definition proposed by Kilteni *et al*.^22^ for the sense of embodiment in virtual reality, and ownership, agency and location were critical contributors to the sense of embodiment according to evidence provided by Longo *et al*.^44^ for the rubber hand illusion. Ratings of presence were assessed because having a virtual body in a virtual environment presented via an HMD can influence the sense of presence^45–47^.

## Methods

### Participants

**Figure 1:**
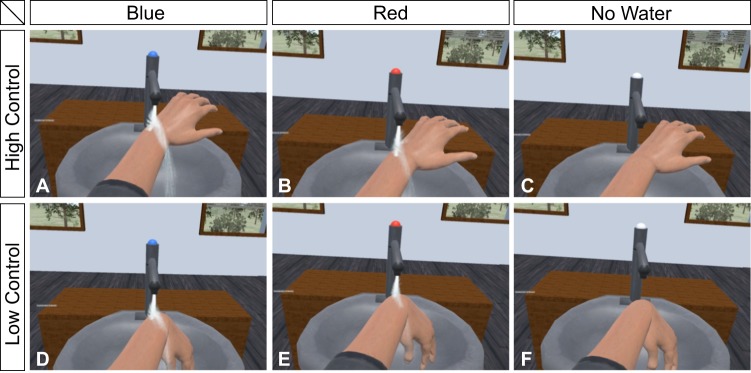
Experimental conditions (screenshots). The panels **A–C** illustrate the three conditions with high control over the virtual hand, subfigures **D–F** the three conditions with low control. In all conditions, participants were instructed to look at the temperature indicator and hold their hands under the virtual tap at the onset of the heat stimulus. The water tap could signal blue (**A,D**), red (**B,E**) or white (**C,F**). In case of no light (white) no water was running when participants held their virtual hands under the water tap.

In a within-subjects design, participants took part in six experimental conditions depicted in Fig. 1. A priori calculation of the optimal sample size to detect at least a moderate effect size (*d* = 0.5) with *α* = 0.05 and a power of 0.90 yielded a sample size of 36. Therefore, we recruited 36 participants via an online database of local study participants. Because two did not comply with task instructions, another two participants were recruited to reach the optimal sample size and allow for a balanced order of task conditions across participants (see below). The 36 participants had a mean age of 24.8 ± 5.4 years (range 18–43, all women, 33 right- and three left-handed). They were compensated for their participation with either 10€ or course credit. None of the participants reported any neurological or psychiatric illness nor acute or chronic pain. None of the participants consumed alcohol or took pain medication 12 hours prior to the start of the experiment. All participants signed informed consent prior to participation in the study that was conducted in accordance with the Declaration of Helsinki. The Ethical Review Board of the Institute of Psychology, University of Würzburg, approved the study protocol.

### Individual heat pain stimulus

Heat stimuli were delivered using a Somedic MSA thermal stimulator (Somedic Sales AB, Hörby, Sweden) and a Peltier thermode with an active surface of 25 × 50 mm attached to the dorsal side of the left forearm, close to the hand.

The stimulus applied during the main experiment had the same temperature in each of the six conditions. There was one heat stimulus per trial. Thermal stimulation started from a baseline temperature 10 °C below the target temperature and heated with 5 °C/s. Once the target temperature was reached, it lasted for 8 seconds. The temperature of the applied heat stimulus was 1 °C above the individual heat pain threshold.

Therefore, we determined the individual pain threshold of each participant before the start of the experiment. For this, participants could increase or decrease the temperature of the thermode in steps of 0.5 °C by button press (starting at a temperature of 37 °C) and should indicate as soon as the heat stimulus felt painful. The average temperature indicated in three runs to be painful was defined as the individual pain threshold temperature (PT). We added 1 °C to this individual PT to ensure that the stimulus would be considered painful^48^. Afterwards the heat stimulus was applied once with the duration of the stimulus to be applied during the main experiments and participants were asked to rate the intensity and unpleasantness of the stimulus on a visual analogue scale ranging from 0 (not painful at all) to 100 (extremely painful). If the intensity rating of the stimulus was in the range between 10 and 90, the determined temperature was selected as the heat stimulus for the main experiment. If it was lower or higher, +1 °C were added or −1 °C subtracted, respectively. For safety reasons, maximum temperature was set to 49 °C.

The mean temperature of the heat stimulus applied during the main experiment was 44.91 °C (*SD* = 1.86). The average intensity rating of the participants was 57.47 (*SD* = 22.77) and the mean unpleasantness rating 50.94 (*SD* = 24.35).

### Setting and virtual reality

Participants were seated throughout the whole experiment. For the main part of the experiment, they wore headphones and a head-mounted display that displayed the virtual environment (HTC Vive, HTC Corp, New Taipei City, Taiwan). The HMD was equipped with a Leap Motion Controller (Leap Motion Inc., San Francisco, California, USA). Participants saw the virtual environment from the perspective of a female avatar, whose location matched their own. The avatar was seated in front of a sink which was placed in the center of a room. The Leap Motion Controller and Assets allowed the participants to move the hands of the avatar in accordance with their own hand and finger movements.

The virtual environment was created in Unity (Unity Technologies, San Francisco, California) and the virtual avatar with Adobe Fuse CC (Adobe Systems Inc., San Jose, California, United States). An asset (Avatar Hand Controller for Leap Motion) was used to control the hands of the avatar and compute arm movements of the avatar based on inverse kinematics.The experimental procedure was programmed using the software Playmaker (HutongGames, LLC) and custom scripts to control heat stimulation and store data.

### Ratings

The main outcome measures of the study were ratings of pain intensity and unpleasantness. After each experimental trial, a visual analogue scale (VAS) ranging from 0 to 100 was displayed in the virtual environment in front of the participants. They could move the slider with the virtual hands and confirm the selected value (displayed above the scale) by selecting the “next” button on the virtual display to answer the next question. The method allowed us to collect the ratings directly after each condition and while participants were still immersed in VR. By using pain ratings, we could use suprathreshold pain stimuli to study pain perception. The anchors for the pain intensity ratings were “not painful at all” and “extremely painful”, for the unpleasantness ratings “not unpleasant at all” and “extremely unpleasant”.

After each experimental trial, we further assessed sense of ownership, sense of agency and sense of self-location with visual analogue scales from 0 to 100. Sense of ownership in our study describes the feeling of mineness towards a body part^22,30^, therefore, participants answered the statement “I had the feeling that the virtual hand was my own” on a scale from “not at all” to “completely”. The anchors remained the same for the following questions. For sense of agency, which is often defined as the experience of initiating and controlling an action^22,30^, the scale read: “I had the feeling of control over the movements of the virtual hands”.

We assessed the extent to which participants had the feeling that the position of the avatar matched the position of their body in space and the extent that participants had the feeling that the positions of the virtual hands matched their own (Location Avatar: “I had the feeling of being in the location of the virtual avatar” and Location Hand: “I had the feeling that the location of the virtual hand matched the location of my hand”)^22^.

Having a virtual body in a virtual environment presented via an HMD can influence the sense of presence^45,46^. The sense of presence can be defined as the sense of “being there” in the virtual environment^49,50^. We assessed presence with the statement “I had the feeling of being present in the virtual world”. A single item to assess (spatial) presence has previously been employed^51,52^ and in the construction of their presence questionnaire Schubert, Friedmann, and Regenbrecht^53^ found that a single item assessing the sense of being there in the virtual environment loaded on all three subscales of their questionnaire and on a general presence factor.

### Post-study questionnaires

To assess symptoms of cybersickness, participants answered the simulator sickness questionnaire (SSQ) following the main experiment^54^. It is a self-report measure that consists of a list of 16 symptoms and participants are asked to indicate the severity level for the individual symptoms on a 4-point scale from “not existing” to “strong”. The questionnaire yields a total score and subscores on nausea, oculomotor symptoms and disorientation. It is an established method to assess symptoms after simulator use and is widely applied in virtual reality research^55–60^.

In a final post-study questionnaire, participants were asked to answer a number of closed and open-ended questions regarding their experience in the virtual environment. These questions were of particular importance to gain insight into the cognitions of the participants during the experiment to aid in the interpretation of the results. The first set of questions were related to their sensations elicited by the virtual water. The first of these questiosn asked participants if they had a particular sensation in response to the virtual water pouring on their virtual hand, they were asked to describe it and indicate if the feeling differed for the experimental conditions. These questions were first posed as open-ended questions in order to reduce expectation/desirability bias. The participants were then asked how often they had this experience on a 5 point scale (1 = in one trial, 2 = in less than half of the trials, 3 = in half of the trials, 4 = in the majority of trials, 5 = in all trials). Next, the questionnaire asked specifically and separately for thermal and tactile sensations with open and closed-ended questions. The participants were further asked what surprised them most about the study, if they experienced discomfort while being immersed in VR (e.g. nausea or other unpleasant feelings) and asked to judge the realness of the virtual environment and the virtual water on separate VAS from 0 (hardly realistic) to 10 (very realistic). For the closed questions, we calculated the percentage of persons that agreed with the respective statements. For the open-ended questions, two independent raters not familiar with the study and its goals decided whether or not the free text answers described specific conditions (experienced thermal sensation, tactile sensation, difference for water conditions and direction of difference: cooling/warming effect in blue (cold water) and red light (warm water) condition and vice versa). In case of differences in their ratings (this was the case for 8% of the ratings), the conflict was resolved by the decision of a third independent rater. We report the number of persons that described their sensations according to the specified conditions.

### Procedure

The experiment started with the determination of the individual heat pain stimulus (one degree above the individual heat pain threshold, as described above). Afterwards, participants were equipped with the head-mounted display and took part in a practice run to accustom them with the virtual environment, the virtual hands and rating scales. All trials (including the practice trial) started with the participants being seated in an upright position with their hands placed on their thighs.

In the practice run, participants were first asked via pre-recorded instructions to look around in the virtual room and perform predefined movements with the virtual hands, they were then informed about the functions of the virtual tap in front of them. They heard the following: “[…] In front of you there is an automatic water tap that is activated as soon as you move your hands underneath. The water temperature is shown via the red and blue display”. They were also informed that sometimes the water tap would not function properly and the temperature indicator would remain white. They were asked to place their arm under the water tap in all conditions and look at their virtual arm/hand. The practice run ended with a practice trial. A water sound recorded from a real water tap was played whenever water was running from the virtual tap. Participants were not informed about the Low Control conditions in advance and at no point during the main study were red and blue explicitly associated with specific temperatures (e.g. warm/hot or cool/cold) in the instructions.

In all conditions, participants were instructed (via pre-recorded instructions) to hold their virtual hand under the water tap at the onset of the heat stimulus and hold it in a position so that the virtual water ran over the location where they felt the heat. They were further instructed to look at the temperature indicator prior to placing their arm under the tap. The temperature indicator changed its colour at the onset of the heat stimulus and remained lit for the duration of the trial. The virtual water started running as soon as the participants placed their hands under the tap. After ten seconds, the end of the heat stimulus, the rating scales (see section *Ratings*) were presented. The next trial began after participants responded to all seven rating scales.

The main study consisted of six experimental conditions depicted in Fig. 1. The temperature indicator could change its colour from white to red or blue, or remain white. In case of a white light, no water would be running from the tap during the trial. Apart from this “Water” manipulation (Blue, Red, No Water), we also manipulated the level of control over the movements of the hand. In the High Control conditions, hand movements of the virtual hands matched those of their real hand. In the Low Control conditions, the hand movements were only updated every other second by activating and deactivating the Leap Motion Controller. This resulted in involuntary movements of the virtual hand as it tilted downwards as depicted in Fig. 1(D–F) as soon as the Leap Motion Controller was deactivated (and was displayed in the current position when it was activated). For each of the six experimental conditions, three trials were presented, resulting in 18 trials in total with a duration of about 20 minutes (depending on the time needed to answer the seven rating scales that were presented after each trial).

The experimental conditions were presented in a pseudo-randomized order such that the order of conditions was balanced across participants. The Water conditions were presented in blocks such that each of the three conditions was presented in pseudo-randomized order for the conditions with high control and low control.

### Hypothesis and statistical analysis

We expected that the blue light condition would reduce pain intensity and unpleasantness ratings, while the red light condition would increase pain ratings compared to the no water condition, respectively, based on top-down modulation of pain. We hypothesised that the High Control condition would result in higher sense of agency ratings compared with the Low Control condition and that the resulting difference in the sense of agency might influence pain ratings.

Separate 2 × 3 repeated measures analysis of variance (rmANOVA) with the factors Water (blue light, red light, no water) and Control (High Control, Low Control) were calculated for the pain, agency, ownership, location and presence ratings. In case of significant main effects, post-hoc *t*-tests with Bonferroni correction for multiple comparisons were conducted. We report mean values and standard errors for the individual conditions.

During few trials, no heat stimulus was applied due to technical errors (3.7% of total trials). These trials were excluded from the analysis and the ratings for the remaining trials for that condition averaged.

## Results

### Pain ratings

**Figure 2:**
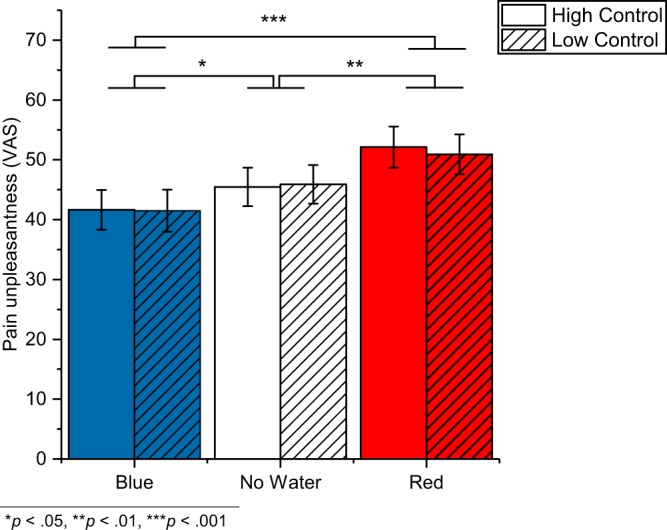
Pain unpleasantness ratings. The graph depicts mean values (±*SE*) for the individual conditions.

**Figure 3:**
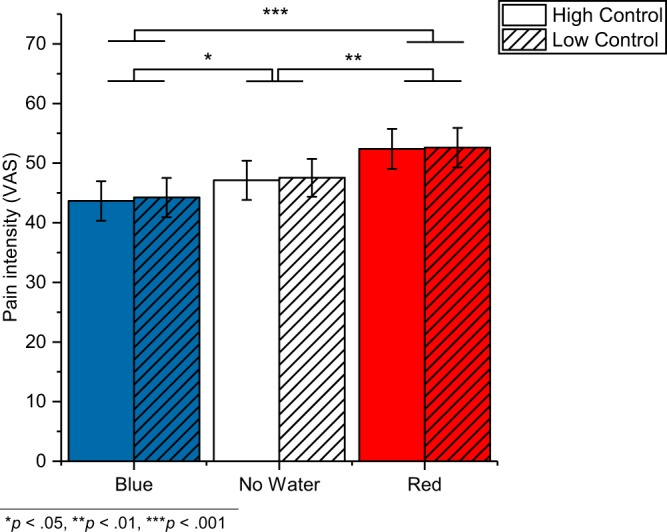
Pain intensity ratings. The graph depicts mean values (±*SE*) for the individual conditions.

Figures 2 and 3 depict pain unpleasantness and pain intensity ratings, respectively, for the experimental conditions. Ratings of individual participants are listed in Supplementary Table S1.

Water had an effect on pain unpleasantness (*F*_2,70_ = 15.57, *p* < 0.001, ${\eta }_{p}^{2}$ = 0.308) and pain intensity (*F*_1.7, 60.2_ = 17.90, *p* < 0.001 GG corrected, ${\eta }_{p}^{2}$ = 0.338). Unpleasantness ratings compared to the no water condition (45.7 ± 3.1) were reduced in the blue light (41.6 ± 3.3; *p* = 0.039) and increased in the red light condition (51.5 ± 3.3; *p* = 0.005), and similarly, the intensity ratings compared to the no water condition (47.3 ± 3.1) were increased in the red light condition (52.5 ± 3.2; *p* = 0.001) and decreased in the blue light condition (43.9 ± 3.2; *p* = 0.033).

Control did neither affect pain unpleasantness (*F*_1,35_ = 0.09, *p* = 0.764, ${\eta }_{p}^{2}$ = 0.003) nor pain intensity (F_2,35_ = 0.15, *p* = 0.705, ${\eta }_{p}^{2}$ = 0.004). And there were no interaction effects (unpleasantness: *F*_2,70_ = 0.22, *p* = 0.801, ${\eta }_{p}^{2}$ = 0.006; intensity: *F*_1.9, 65.7_ = 0.01, *p* = 0.988 GG corrected, ${\eta }_{p}^{2}$ < 0.001).

### Sense of ownership

The Control manipulation significantly affected the ownership ratings (*F* _1,35_ = 15.73, *p* < 0.001, ${\eta }_{p}^{2}$ = 0.310) which were higher in the High Control (64.7 ± 2.6) as compared to the Low Control (54.8 ± 3.1) condition. Water affected the ownership ratings (*F* _1.6, 56.6_ = 3.89, *p* = 0.034, GG corrected, ${\eta }_{p}^{2}$ = 0.100) too, however, the post-hoc comparisons were not significant (all *p* > 0.05; blue light: 60.5 ± 2.7, red: 61.1 ± 2.7, no water: 57.8 ± 2.7). There was no interaction effect, *F* _2, 69.5_ = 1.54, *p* = 0.223 GG corrected, ${\eta }_{p}^{2}$ = 0.042.

### Sense of agency

**Figure 4:**
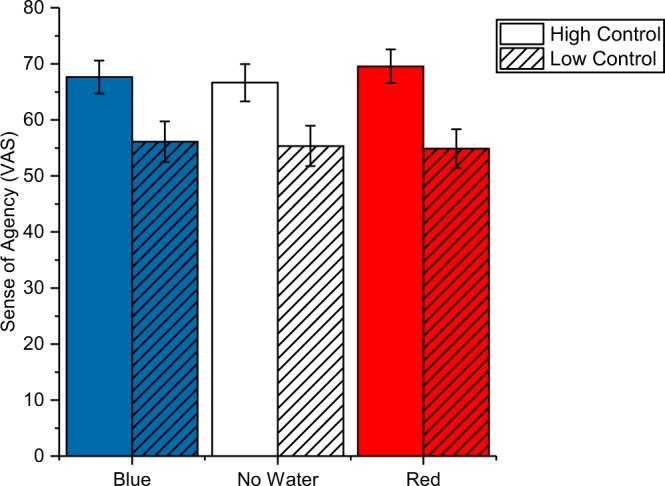
Sense of agency ratings. The graph depicts mean values (±*SE*) for each experimental condition. The rmANOVA revealed a main effect of condition (high vs. low control) demonstrating that the experimental manipulation was successful.

The Control manipulation had a significant effect on the sense of agency (*F* _1,35_ = 19.18, *p* < 0.001, ${\eta }_{p}^{2}$ = 0.354) which was higher in the High Control (67.9 ± 2.8) as compared to the Low Control (55.4 ± 3.4) condition. Sense of agency rating for each condition are depicted in Fig. 4. The Water manipulation did not affect the sense of agency ratings, *F* _2,70_ = 0.41, *p* = 0.668, ${\eta }_{p}^{2}$ = 0.011. There was no interaction effect, *F* _2,70_ = 0.80, *p* = 0.452, ${\eta }_{p}^{2}$ = 0.022.

### Sense of presence and location

The factor Control had a significant effect on the presence ratings (*F* _1,35_ = 7.46, *p* = 0.010, ${\eta }_{p}^{2}$ = 0.176) as participants felt slightly more present in the virtual world in the High Control (71.0 ± 2.9) as compared to the Low Control (66.3 ± 3.3) condition. The factor Water did not affect presence ratings, *F*_2,70_ = 1.10, *p* = 0.340, ${\eta }_{p}^{2}$ = 0.030, and there was no interaction effect, *F*_2,70_ = 1.19, *p* = 0.312, ${\eta }_{p}^{2}$ = 0.033.

The factor Control had a significant effect on the location ratings for the hand (*F*_1,35_ = 21.84, *p* < 0.001, ${\eta }_{p}^{2}$ = 0.384) and the avatar (*F*_1,35_ = 12.48, *p* = 0.001, ${\eta }_{p}^{2}$ = 0.263). The location of the virtual hand was rated to match the location of the real hand more closely in the High Control (69.2 ± 2.8) as compared to the Low Control (55.4 ± 3.4) condition. Water affected the location ratings (*F*_2,70_ = 5.76, *p* = 0.005, ${\eta }_{p}^{2}$ = 0.141) too, with a greater rated match in the blue light (63.4 ± 2.9) and red light conditions (63.7 ± 2.7) as compared to the no water (59.8 ± 2.8) condition (*p* = 0.033 and *p* = 0.018). There was no interaction effect, *F*_2,70_ = 2.01, *p* = 0.142, ${\eta }_{p}^{2}$ = 0.054.

Similarly, the location of the virtual avatar was rated to match the location of the real body more closely in the High Control (69.5 ± 2.8) as compared to the Low Control (62.8 ± 3.1) condition. Water affected the location ratings (*F*_1.5, 53.4_ = 4.59, *p* = 0.022 GG corrected, ${\eta }_{p}^{2}$ = 0.116) as the virtual avatar was rated to match the location of the real body more closely in the red light condition (67.6 ± 2.7) as compared to the no water (64.1 ± 3.1) condition (*p* = 0.012). There was no interaction effect, *F*_1.9, 67.5_ = 0.30, *p* = 0.732 GG corrected, ${\eta }_{p}^{2}$ = 0.009.

### Post-study questionnaires

#### Sensations elicited by virtual water and coloured lights

Table 1 lists the number of people that described a specific sensation in response to the virtual water pouring on their virtual hand within the post-study questionnaire. Post-experimental assessment indicated that the lights induced a temperature illusion and affected pain perception, i.e., 72% (n = 26) of the participants reported a thermal feeling in response to the virtual water pouring on their virtual hand and they reported effects on their pain perception in about half the runs (rating of *M* = 3.5, *SD* = 1). Most participants (*N* = 25) indicated that they experienced a difference between the conditions and a large number of participants indicated that the blue, “cold condition” had a cooling effect and the “warm/hot condition” a worsening effect on their heat pain. Additional tactile feelings were reported by 58% of the participants (n = 21) for about half the runs (*M* = 2.6, *SD* = 1.3). The free text answers indicate that two participants (02 and 36) experienced paradoxical sensations in some runs in response to the virtual water. Participant 02 reported that the heat pain felt warmer in the blue light condition and participant 36 reported a sensory conflict, because the heat did not decrease despite the “cold water”. We did not specifically ask for this in the questionnaire, but one participant alluded to a difference between the control conditions, naming it as a prerequisite that the virtual hand was “under control” to feel a cooling effect in the blue and a warming effect in the red light condition.

**Table 1:** Post-study questionnaire items in relation to sensations elicited in response to the virtual water pouring on the virtual hand.

Questionnaire Item	Number of participants
**Sensation in response to virtual water**	**Yes: 31, No: 5 (86%)**
*Free text description of sensation*:	
*Thermal*	24
*Tactile*	9
*Generally cooler*/*pain alleviating*	11
*Generally warmer*/*worsening of pain*	4
*Difference between conditions*	14
*Warmer*/*less pleasant in red light condition*	8
*Cooler in red light condition*	0
*Cooler*/*more pleasant in blue light condition*	11
*Warmer in blue light condition*	0
**Difference between red and blue light**	**Yes: 25** (out of 31)
*Free text description of sensation:*	
*Cooler*/*more pleasant in blue light condition*	17
*Warmer*/*less pleasant in red light condition*	11
*Paradoxical sensation*/*other*	6
**Thermal sensation in response to virtual water**	**Yes: 26, No: 10** (72%)
**Thermal difference between red and blue light**	**Yes: 20** (out of 26)
*Free text description of sensation*:	
*Cooler*/*more pleasant in blue light condition*	16
*Warmer*/*less pleasant in red light condition*	9
*Paradoxical sensation*/*other*	5
**Tactile sensation in response to virtual water**	**Yes: 21, No: 15** (58%)

The closed-ended questionnaire items are printed in bold and the categorized free text answers to the open-ended questions are set in italics.

#### Realness of virtual reality and cybersickness

In response to the question what was most surprising about the study, most comments concerned the hand movements and the realness of the virtual environment. Participants were positively surprised how well the movements of the virtual hands matched their real movements and that they could interact with the virtual environment using their hands. On the VAS (range 0–10), the degree of realness of the virtual environment was rated as *M* = 6.8 (*SD* = 2.1) and the realness of the virtual water as *M* = 6.1 (*SD* = 2.1). Many participants also commented on the sensations elicited by the virtual water. In response to the question about sickness or feelings of discomfort while being immersed in the virtual environment, only one participant indicated that she had slightly blurred vision toward the end of the experiment.

#### Simulator sickness questionnaire

The mean total score of the SSQ of 20.57 (*SD* = 18.16) indicated only mild side effects with the nausea subscale having the lowest mean (*M* = 9.01; *SD* = 13.29). Fatigue was the most frequently reported symptom (*n* = 22), followed by eyestrain (*n* = 18) and difficulty focusing (*n* = 17), blurred vision and fullness of head (each *n* = 12). Most of the symptoms were rated as mild and none as strong.

### Explorative analysis

**Figure 5:**
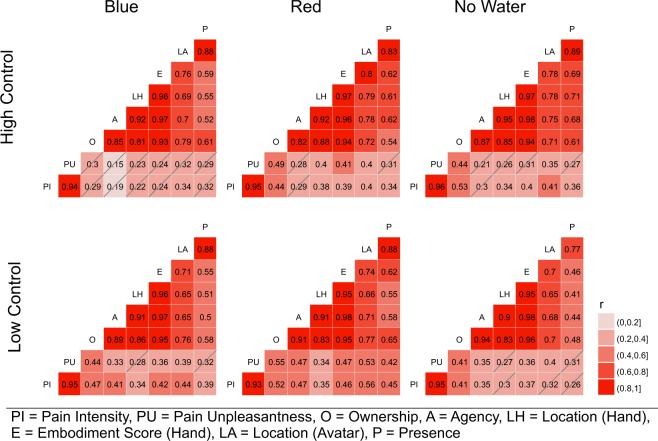
Correlations (Pearson’s *r*) for all items that were answered after each trial for each experimental condition. Because of the strong intercorrelations of the sense of ownership, sense of agency and sense of location (hand) and to facilitate interpretation of the data, we computed an embodiment score for the hand consisting of the average score of the three items (O, A, LH). Non-significant correlations (*p* > 0.05) are crossed out. If correcting the alpha level for multiple comparisons per condition (0.05/28), all strong correlations (> 0.5) remain significant.

To explore how the sense of ownership affected pain ratings, we calculated correlations (Pearson’s *r*) with pain ratings and found medium to large effects (*r* = 0.30 to 0.55). See Fig. 5 for correlations between pain ratings, sense of ownership, sense of agency, sense of location (hand, avatar) and presence ratings.

## Discussion

This study demonstrated that context manipulations in an immersive virtual reality and interaction with this virtual world via embodied virtual hands have a modulatory effect on experimentally induced pain likely due to top-down effects on pain processing based on previous experiences. Specifically, the context manipulations of a red or blue light while virtual water was running over the participants’ virtual hands caused increased or decreased pain ratings (intensity and unpleasantness), respectively, in response to a thermal pain stimulus. We argue that the context manipulation successfully induced a temperature/water illusion as most participants associated the blue light with cool/cold temperatures and the red light with warm/hot temperatures and most reported a thermal sensation in response to the virtual water running over their virtual hands (72%). This temperature illusion exerted a rather large top-down modulation on the processing of the simultaneously applied thermal pain, i.e., warm or cold temperature illusions facilitated or inhibited the processing of the thermal pain stimulus.

The study suggests that context manipulation within an embodied setting in immersive virtual environments can have a strong effect on pain and should be considered within VR based pain treatments. Mean differences between experimental conditions on the VAS pain ratings (0–100) of up to 10 points on the group level and up to 41 points for individual participants are in the range that has previously been suggested to mark a clinically meaningful change in studies with pain patients^61,62^. Our effects were demonstrated with healthy participants under experimental conditions; hence, further research needs to establish whether similar effects can be obtained for acute (and chronic) pain patients.

In a previous study, Martini *et al*.^8^ manipulated the skin colour of a virtual hand, but found only a small effect on pain. A manipulation that more closely resembles real life experiences, as in our paradigm, in which the VHI is induced by co-located movements of the virtual and real hands, and the context manipulation builds upon previous experiences in daily life are possible reasons for the stronger top-down modulation of pain. Another important factor are the different types of heat stimulation and outcome measures, i.e. pain thresholds vs. pain ratings.

Research on placebo effects on pain suggest that expectations and/or conditioning effects are crucial for the observed top-down influences^48,63,64^. On the one hand, based on previous experiences the virtual colours may cause specific expectations which modulate pain processing. On the other hand, the colours blue and red may have become conditioning stimuli, which automatically elicit responses that modulate pain processing, again, based on previous experiences. Likely, both processes may have played a role here and are based on ontogenetic experiences as only children older than six years reliably report these associations between blue or red and cold or warm, respectively^4^. Future research is necessary to disentangle the contributions of explicit expectations and implicit conditioning processes on the effects of virtual reality on pain processing.

As reported above, most participants experienced a thermal sensation in response to the virtual water pouring on their hand and anticipated consecutive pain relief or increase. Along with the observed pain modulation, the findings suggest that anticipation is a crucial factor. However, other cognitive factors also play a role, such as experienced sensory conflict. One participant reported to be irritated by the fact that the heat stimulus remained constant in the “cold water” condition and for this participant pain ratings were higher in the blue as compared with the red light condition. Another participant explicitly stated that the heat pain felt warmer in the blue light condition than in the red, nevertheless mean pain ratings were lower in the blue light condition for this participant.

Apart from the context (water) manipulation, we also studied the influence of agency on pain modulation via virtual reality. The results indicate that our agency manipulation was successful, but we were unable to reveal effects on pain ratings. Nevertheless, even stronger agency manipulations might have an effect (e.g. high control vs. no control instead of low control). Furthermore, we focused mainly on the experience of controlling the virtual hand and used explicit measures of agency. It might be worthwhile to draw upon/implement intentional binding paradigms^65^ in embodied virtual reality scenarios to assess implicit measures of agency and investigate factors such as outcome choice and initiation of motor actions^66,67^. For instance, pain ratings could be lower (sensory attenuation greater) with control over the outcome^66^.

The agency manipulation of our study positively influenced sense of ownership, sense of location (for the virtual avatar and the virtual hand) and presence. These results are in line with findings from previous VHI studies and a study incorporating intentional binding into the RHI also revealing positive correlations between objective agency and sense of ownership^40,68^. We conclude that these factors interact to produce ownership illusions, and would like to stress the importance of bottom-up factors in creating ownership illusions in virtual reality^40^. The high average ownership ratings in all conditions indicate that the participants felt as if the virtual hand was (to a certain degree) their hand and, therefore, a virtual hand illusion was achieved.

Strong intercorrelations between the sense of ownership, sense of agency and sense of location strengthen claims that they can be considered as key aspects of the sense of embodiment in virtual reality^22^. It has previously been proposed that embodiment plays an important role for the feeling of presence^45^. Our findings contribute to this line of research by demonstrating that if an avatar is displayed in the position of the user and hand and arm movements are mapped according to real movements, even relatively small changes as part of the experimental manipulation (high vs. low agency) can influence the sense of presence. The effect of embodiment on presence ratings can also be seen in the high correlations of our explorative analysis.

It is an established finding that looking at the own painful body part can have an analgesic effect^69–72^. Several studies investigated if this visually induced analgesia can also be elicited through ownership over an artificial body part, e.g. a rubber hand^73–78^, a virtual hand^8,35,36,79^ or virtual legs^80^. Most of these studies focused on the visual appearance and found that the vision of an “owned” artificial limb can have an analgesic effect, albeit a small one, but that this effect can be conversed if the artificial hand looks injured or is unnaturally bent^77,81^. The explorative analysis of our study revealed moderate to strong positive correlations between ownership and pain ratings. This indicates that the stronger the perceived ownership over the hand the more intense and unpleasant the perceived pain. This finding has not previously been reported and seems to contradict the results from previous studies. Martini *et al*.^81^, however, argued that in some cases (e.g. if the location of the fake and real body do not match) the analgesic effect of the vision of one’s “own” body is rather due to attentional mechanisms or disownership of the real body. Following this line of thought, it is likely that the analgesic effects of distraction^2,15,16,82^ are less strong if the experience in virtual reality becomes more lifelike. Biocca^45^ described it as the “cyborg’s dilemma” that “the more natural the interface the more “human” it is, the more it adapts to the human body and mind. The more the interface adapts to the human body and mind, the more the body and mind adapts to the non-human interface”. We implemented a virtual hand illusion that allows natural interaction with the virtual world. However, with increasing ownership over a virtual body that is integrated into the bodily self-model, other beneficial (and stronger) analgesic effects, most importantly distraction, might diminish. This raises the question under which conditions virtual embodiment is beneficial for VR based treatment of pain. For healthy participants, for instance, Martini *et al*.^8^ found the highest pain threshold for the condition in which participants focused on a spot next to their virtual hand, instead of a spot on their hand, where they received a painful stimulus. This finding could support the assumption that the effect of distraction is larger than the analgesic effect of looking at one’s own body. For patients with painful body conditions, however, virtual embodiment of a healthy-looking/moving virtual hand could be beneficial^39,83^ and future research needs to investigate the specific conditions under which these effects go beyond the known effect of VR distraction^16^.

Some limitations of the current study need to be addressed. We demonstrated these effects with a female population, which is important as women usually report more severe pain, more frequent and longer lasting pain as compared to men^84^, but the effects remain to be demonstrated for men. In our study we employed continuous, single item measures for embodiment (ownership, agency, location) and presence. This allowed participants to rate the aspects after every trial while they were still immersed in VR, however, we did not use multi-item ratings per study construct, therefore limiting comparisons with previous studies. Investigating the underlying neural processes for the observed effect would shed further light on the mechanisms involved in the modulation of pain. The discussed mechanisms for the top-down modulation of pain (anticipation of pain relief or intensification and attentional mechanisms) have previously been associated with different descending modulatory systems^82^. And lastly, as mentioned above, future studies should consider stronger agency manipulations.

In conclusion: The study demonstrates a strong modulatory effect of context manipulation within an embodied setting in immersive virtual reality on pain perception. Clinically meaningful effects for individual participants suggest that these effects should be considered for virtual reality based treatments of acute pain.

No effects of agency on pain perception were revealed by the study. However, strong correlations were revealed for ratings of embodiment (sense of ownership, sense of agency, sense of location) and the sense of presence. Moderate to strong correlations between ownership and pain ratings suggest that factors related to virtual embodiment influence pain perception – these factors need to be investigated further.

## Supplementary information

Supplementary Table S1

## Data Availability

The pain ratings of individual participants are listed in Supplementary Table S1. The datasets analysed during the current study are available from the corresponding author on reasonable request.
