# Supplementary material for: Heat pain modulation with virtual water during a virtual hand illusion
Source: Sci Rep. 2019 Dec 13;9:19137. doi: 10.1038/s41598-019-55407-0 (PMC6911006; doi:10.1038/s41598-019-55407-0)
Supplement: Supplementary file 1 — Supplementary Table S1 [file 41598_2019_55407_MOESM1_ESM.pdf]

Supplementary Information

Heat pain modulation with virtual water during a virtual hand illusion

Ivo Käthner*[1], Thomas Bader[1] & Paul Pauli[1,2]

[1]Department of Psychology I, Biological Psychology, Clinical Psychology and Psychotherapy, University of Würzburg, Würzburg, Germany

[2]Center of Mental Health, Medical Faculty, University of Würzburg, Würzburg, Germany

Supplementary Table S1. Individual pain ratings (intensity and unpleasantness) for each of the six experimental conditions.

	High Control (HC)						Low Control (LC)						Intensity (HC+LC)			Unpleasantness (HC+LC)		
Participant	Blue_Int	Blue_Unpl	Red_Int	Red_Unpl	No_Int	No_Unpl	Blue_Int	Blue_Unpl	Red_Int	Red_Unpl	No_Int	No_Unpl	Blue_Int	Red_Int	No_Int	Blue_Unpl	Red_Unpl	No_Unpl
1	57.33	56.00	51.00	46.67	42.33	41.33	53.00	45.00	53.00	47.50	56.67	55.00	55.17	52.00	49.50	50.50	47.08	48.17
2	28.33	20.00	37.67	27.67	33.33	22.00	22.33	17.67	27.00	20.00	19.50	17.50	25.33	32.33	26.42	18.83	23.83	19.75
3	41.00	45.00	49.67	55.67	55.67	61.00	41.67	41.33	54.67	56.00	50.67	54.33	41.33	52.17	53.17	43.17	55.83	57.67
4	18.00	21.33	59.00	68.00	48.67	44.67	45.00	32.50	61.67	68.00	34.67	23.67	31.50	60.33	41.67	26.92	68.00	34.17
5	56.00	55.67	55.33	59.00	51.67	49.00	58.33	63.00	50.00	51.00	58.67	56.00	57.17	52.67	55.17	59.33	55.00	52.50
6	23.67	16.00	25.00	26.00	21.33	21.33	23.00	7.33	20.67	13.33	21.00	23.33	23.33	22.83	21.17	11.67	19.67	22.33
7	68.00	61.33	66.33	54.00	60.67	57.00	62.33	52.33	62.67	55.00	58.67	50.33	65.17	64.50	59.67	56.83	54.50	53.67
8	58.00	51.00	74.67	83.00	76.67	74.67	75.67	69.00	83.33	76.67	78.00	70.33	66.83	79.00	77.33	60.00	79.83	72.50
9	52.33	52.00	67.50	68.50	50.50	56.00	38.67	35.33	52.67	51.00	37.67	32.33	45.50	60.08	44.08	43.67	59.75	44.17
10	32.67	25.67	30.00	34.00	31.33	32.67	26.00	21.33	30.00	37.67	29.00	28.67	29.33	30.00	30.17	23.50	35.83	30.67
11	54.67	70.67	53.00	70.67	29.67	37.33	41.33	54.67	46.00	67.67	47.67	53.00	48.00	49.50	38.67	62.67	69.17	45.17
12	57.67	49.00	73.00	70.00	60.33	54.33	53.67	47.00	64.33	59.33	72.50	70.00	55.67	68.67	66.42	48.00	64.67	62.17
13	9.00	9.67	16.50	18.33	15.00	17.67	9.67	12.67	27.00	19.00	15.33	15.67	9.33	21.75	15.17	11.17	18.67	16.67
14	18.33	2.67	36.00	29.00	22.50	14.50	24.33	14.00	40.33	26.33	37.00	22.00	21.33	38.17	29.75	8.33	27.67	18.25
15	50.67	34.67	65.00	61.00	74.00	63.33	43.33	33.00	72.33	68.33	53.67	44.00	47.00	68.67	63.83	33.83	64.67	53.67
16	59.33	61.00	76.00	68.33	73.33	69.00	77.67	71.67	82.50	72.00	74.67	72.33	68.50	79.25	74.00	66.33	70.17	70.67
17	43.67	50.33	69.33	81.33	46.67	43.67	54.00	52.67	65.67	65.00	57.33	60.33	48.83	67.50	52.00	51.50	73.17	52.00
18	47.00	40.33	60.33	62.33	56.67	51.33	45.33	49.33	57.33	56.00	49.67	49.33	46.17	58.83	53.17	44.83	59.17	50.33
19	11.67	17.67	34.00	40.00	9.67	10.33	13.00	15.67	21.67	25.67	7.50	11.00	12.33	27.83	8.58	16.67	32.83	10.67
20	60.33	56.00	52.67	55.33	58.67	54.00	60.33	55.67	57.67	53.67	57.00	52.00	60.33	55.17	57.83	55.83	54.50	53.00
21	79.33	71.67	81.00	73.33	66.33	49.00	78.67	81.33	76.33	85.33	73.33	80.00	79.00	78.67	69.83	76.50	79.33	64.50
22	14.67	21.67	31.33	40.67	24.00	26.33	12.33	21.00	14.00	23.67	29.00	35.50	13.50	22.67	26.50	21.33	32.17	30.92
23	10.00	12.00	8.67	3.67	15.33	13.67	11.00	4.00	14.33	9.67	25.50	19.00	10.50	11.50	20.42	8.00	6.67	16.33
24	43.00	37.67	48.67	49.33	39.00	46.00	30.33	29.33	46.67	47.33	35.00	40.33	36.67	47.67	37.00	33.50	48.33	43.17
25	61.33	63.67	54.67	51.33	71.00	70.00	64.00	57.00	75.33	67.00	67.33	64.33	62.67	65.00	69.17	60.33	59.17	67.17
26	11.00	14.33	9.00	10.00	30.67	30.00	13.67	11.67	43.33	42.00	28.33	32.33	12.33	26.17	29.50	13.00	26.00	31.17
27	37.33	34.33	63.33	62.67	48.33	44.00	37.33	27.67	65.67	67.67	51.67	51.67	37.33	64.50	50.00	31.00	65.17	47.83
28	31.33	33.33	73.00	71.33	43.00	49.67	51.67	43.00	60.67	67.33	60.00	73.67	41.50	66.83	51.50	38.17	69.33	61.67
29	41.67	32.67	39.00	34.00	47.67	49.00	56.00	55.33	44.00	34.00	37.33	31.67	48.83	41.50	42.50	44.00	34.00	40.33
30	39.00	37.00	35.00	31.33	38.00	31.33	36.33	35.33	31.33	31.00	20.67	25.00	37.67	33.17	29.33	36.17	31.17	28.17
31	38.67	41.33	44.67	39.00	19.67	15.33	38.67	30.00	53.67	46.67	46.33	32.33	38.67	49.17	33.00	35.67	42.83	23.83
32	55.00	51.67	57.00	46.00	56.00	54.50	48.67	48.33	62.00	51.00	64.33	58.67	51.83	59.50	60.17	50.00	48.50	56.58
33	59.00	47.67	63.33	62.00	61.00	60.67	54.00	46.67	47.00	50.00	60.50	49.00	56.50	55.17	60.75	47.17	56.00	54.83
34	83.33	83.00	83.67	84.00	80.67	82.00	68.33	76.00	82.67	71.67	68.33	67.67	75.83	83.17	74.50	79.50	77.83	74.83
35	54.00	50.00	82.00	75.67	73.00	67.67	52.33	59.00	82.00	78.33	54.33	54.00	53.17	82.00	63.67	54.50	77.00	60.83
36	64.67	71.00	59.33	63.67	63.67	73.33	70.33	76.67	64.50	71.00	72.67	76.00	67.50	61.92	68.17	73.83	67.33	74.67
